# Bringing Community and Academic Scholars Together to Facilitate and Conduct Authentic Community Based Participatory Research: Project UNITED

**DOI:** 10.3390/ijerph13010035

**Published:** 2015-12-22

**Authors:** Dwight Lewis, Lea Yerby, Melanie Tucker, Pamela Payne Foster, Kara C. Hamilton, Matthew M. Fifolt, Lisle Hites, Mary Katherine Shreves, Susan B. Page, Kimberly L. Bissell, Felecia L. Lucky, John C. Higginbotham

**Affiliations:** 1 Institute for Rural Health Research, The University of Alabama, Tuscaloosa, AL 35487, USA; LEWIS060@cba.ua.edu (D.L.Jr.); YERBY002@cchs.ua.edu (L.Y.); PPAYNE-FOSTER@cchs.ua.edu (P.P.F.); KHAMILTON1@crimson.ua.edu (K.C.H.); SPAGE@cchs.ua.edu (S.B.P.); 2 Department of Community and Rural Medicine, The University of Alabama, Tuscaloosa, AL 35487, USA; 3 Department of Family Medicine, The University of Alabama, Tuscaloosa, AL 35487, USA; MTUCKER@cchs.ua.edu; 4 Evaluation and Assessment Unit, The University of Alabama at Birmingham, Birmingham, AL 35294, USA; MFIFOLT@uab.edu (M.M.F.); LHITES@uab.edu (L.H.); 5 Department of Health Care Organization and Policy, The University of Alabama at Birmingham, Birmingham, AL 35294, USA; 6 Institute for Communication and Information Research, The University of Alabama, Tuscaloosa, AL 35487, USA; MKALSIP@ua.edu (M.K.S.); KBISSELL@ua.edu (K.L.B.); 7 Black Belt Community Foundation, Selma, AL 36701, USA; FJONES@blackbeltfound.org

**Keywords:** community based, participatory, collaboration, cultural competency, grantsmanship, research literacy, rural, obesity

## Abstract

Cultural competency, trust, and research literacy can affect the planning and implementation of sustainable community-based participatory research (CBPR). The purpose of this manuscript is to highlight: (1) the development of a CBPR pilot grant request for application; and (2) a comprehensive program supporting CBPR obesity-related grant proposals facilitated by activities designed to promote scholarly collaborations between academic researchers and the community. After a competitive application process, academic researchers and non-academic community leaders were selected to participate in activities where the final culminating project was the submission of a collaborative obesity-related CBPR grant application. Teams were comprised of a mix of academic researchers and non-academic community leaders, and each team submitted an application addressing obesity-disparities among rural predominantly African American communities in the US Deep South. Among four collaborative teams, three (75%) successfully submitted a grant application to fund an intervention addressing rural and minority obesity disparities. Among the three submitted grant applications, one was successfully funded by an internal CBPR grant, and another was funded by an institutional seed funding grant. Preliminary findings suggest that the collaborative activities were successful in developing productive scholarly relationships between researchers and community leaders. Future research will seek to understand the full-context of our findings.

## 1. Introduction

### 1.1. Obesity and Rurality

Obesity remains a public health challenge for the US [1], and specifically the US Deep South, as the obesity prevalence of this region is well over 30% [2]. Recent population level estimates suggest that both African American youth and adult women are more likely to be obese compared to their White counterparts [1]. Furthermore, cross-sectional annual trends in the US propone that obesity risk is increasing among African American men [3]. Evidence also suggest that obesity is not only associated with increased odds of acquiring co-morbid chronic disease conditions [4], but is also associated with poorer quality of life [5] and potentially poses as a significant economic burden for the individual and global economy [6]. As such, interventions aimed at reducing racial obesity-related disparities may have conferred benefits beyond that of physiological health.

The relationship between disadvantaged sociological conditions and obesity-related morbidities is an ongoing concern [7,8]. Though individual socioeconomic characteristics are associated with obesity [9], evidence also suggests that the conditions of communities’ built environment are significantly associated with obesity [10]. Accordingly, it is not surprising that population level estimates suggest adult rural residents in the US are at greater risk for obesity than their urban counterparts [11]. The true causative factors of this disparity are debatable due to the complex etiology of obesity [12] and an overreliance on self-reported approaches (e.g., dietary behaviors [13] and physical activity [14]) in research investigating comparable health disparity matters [15,16]. However, it is difficult to overlook the historical presence of poverty [17] and the lack of health care access [18] within rural communities. Hence, given rural America’s sociological challenges, interventions tailored for rural communities’ structured and sociopolitical environment appears to be a plausible approach to improving rural population health.

### 1.2. Community Based Participatory Research

Community based participatory research (CBPR) is recognized as an umbrella of methodologies centered on equity between academic and non-academic stakeholders throughout the development, implementation, and dissemination phases of research designed to lead to an improved society [19,20]. The public health advantages associated with CBPR are well documented in the literature [19,20,21], as the science behind CBPR in health research has progressed tremendously over the past 20 years. Though many CBPR projects are designed to address health outcomes directly, it is important not to discount the process of empowering community members to proactively address challenging circumstances (e.g., health inequities). Current literature suggests that building community capacity through empowerment is essential for interventions having a sustainable effect on health disparities [22]. Therefore, if a community is unable to collectively sustain a health intervention or develop new health interventions once the researcher has left, then it is debatable whether that particular CBPR partnership was successful despite any potential health improvements participants experienced while the researcher was present.

That said, achieving equal participation between academic and non-academic stakeholders is very difficult given: (1) time constraints for many funded CBPR projects; (2) varying levels of research expertise between community leaders; and (3) cultural competency among many academic researchers [23], all contributing to a literature that is inundated with community interventions cloaked as CBPR [24]. One approach to address these challenges is to offer training to both academic and non-academic stakeholders in the areas in which they are unfamiliar so that knowledge barriers that might hinder effective communication are narrowed. Such training could empower community members to become active participants in the improvement of population health, and could conversely provide academic researchers with a better understanding of the communities and cultures in which they are working with.

### 1.3. Project UNITED

Project UNITED (Using New Interventions Together to Eliminate Disparities) is a comprehensive CBPR initiative with a dual purpose. First, Project UNITED aspires to empower communities to take a leadership role in scholarly activities designed to ameliorate health concerns within their community. Second, Project UNITED serves as a community engagement hub for both academic and non-academic stakeholders who desire to improve population health in rural Alabama. More specifically, Project UNITED is a collaboration between The University of Alabama and the Black Belt Community Foundation supporting scholarly partnerships between rural predominantly African American Black Belt communities, academic researchers, and community medical professionals. Project UNITED seeks to develop and expand participatory practices to contribute to the evidence-base of community engagement to improve health within rural populations.

Project UNITED is currently completing its pilot phase, facilitating the formation of partnerships between academic and non-academic stakeholders. Partners work together to develop fundable projects designed to abate obesity and related diseases in Alabama’s Black Belt region. In addition to sustainability activities, Project UNITED will begin to implement multilevel interventions across the Black Belt region through the use of the infrastructure it designed during its pilot phase. Once renowned for its lucrative black topsoil in an era when agriculture dominated the US economy, the predominately rural Black Belt region of Alabama is now better known for its educational, poverty, and health care access disparities [25]. As such, Alabama’s Black Belt region serves as the epicenter for health and economic transformation for state improvements.

As an integral component of Project UNITED, collaborative academic and non-academic partners submit a research proposal to a Community Advisory Board (CAB), which is a community-oriented research oversight advisory board comprised of leaders from the Black Belt community. Project UNITED’s CAB serves as the “balance of power” between academic and non-academic stakeholders to ensure that parity of control is present among current and future CBPR projects. Though most CBPR research grant proposals submitted by participants to date through Project UNITED’s assistance were projects initially submitted for a competitive internal grant, it is Project UNITED’s long-term goal for this review process to serve as a resource for all CBPR research projects within the Black Belt community.

**Figure 1:**
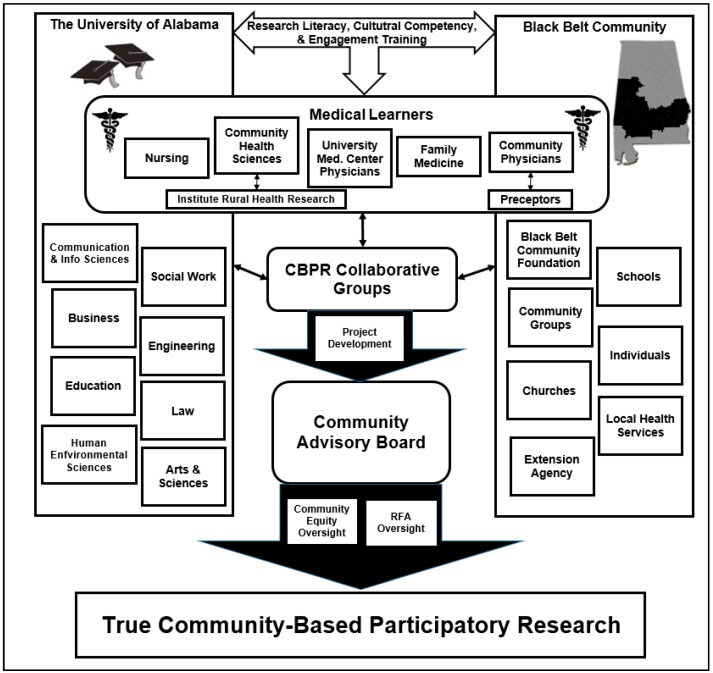
Project Using New Interventions Together to Eliminate Disparities (UNITED) organizational structure.

What distinguishes Project UNITED from standard research centers are the activities that occur before the submission of a CBPR grant application. The purpose of this manuscript is to highlight CBPR activities implemented in Project UNITED, including: (a) a summary of the development of Project UNITED’s pilot grant request for application (RFA); (b) engagement and research training activities to facilitate the submittal of a grant proposal by academic and non-academic scholars serving as equitable principal investigators (PIs); and (c) a brief discussion of grant funded projects designed by participants of Project UNITED’s initial cohort. A visual of Project UNITED’s organizational structure is displayed in Figure 1. All activities discussed in this manuscript were approved by an accredited academic institutional review board and measured using validated practices in evaluation and assessment techniques.

## 2. Experimental Section: CBPR Approaches

### 2.1. Community Participatory Request for Application (RFA)

Little exists in the literature providing insights into the development and awarding of CBPR grants/requests for applications (RFAs). Many published CBPR studies were funded as pilot grants, and little is known about the community’s involvement during the creation of the RFAs that fund these published CBPR studies. Given existing challenges in CBPR research [26,27], it is essential to ensure opinions and recommendations of community members are involved in the process of developing community-oriented pilot grants. Integrating the community into CBPR RFA development and award process will better integrate the target community’s ideals into the grant’s purpose and evaluative criteria. Accordingly, Project UNITED integrated the CAB into the internal pilot grant RFA, to build trust between The University of Alabama’s administrators and the Black Belt Community Foundation, as trust between researchers and community members is an important component of a successful CBPR relationship [28]. By increasing the level of trust between researchers and the community-at-large, it was hoped that the time to build a successful CBPR relationship would be expedited, addressing one key constraint identified as a barrier to a successful CBPR projects [29].

The development of Project UNITED’s CBPR pilot grant RFA started with a search for existing community RFAs and pilot CBPR RFAs. Though the initial online search produced a large sample of RFAs, the publicly available RFAs did not provide sufficient details about the application’s review process and community members’ role during that review process. This led to phone calls and email communications with regional Alabama non-profit organizations that annually fund community projects. As a result of these communications, Project UNITED acquired multiple regional community-oriented RFAs and information regarding how local non-profit organizations review grant applications.

Next, Project UNITED and its CAB met and reviewed the RFA examples, as well as local county health assessment data related to obesity. A review of the local county health assessment data provided Project UNITED’s academic investigators an opportunity to highlight obesity as a public health issue in Alabama’s Black Belt to community leaders, resulting in consensus between the two groups that obesity would be the focus of the pilot RFA. The CAB verbally expressed that it wanted the composition of the RFA to be externally perceived as “scientifically rigorous”, but not so rigid that it would intimidate prospective non-academic community scholars. Also, the CAB wanted a portion of the pilot grant’s budget earmarked for tangible deliverables to the community and an administrative cap for funds designated toward investigators’ salary and institutional overhead. Moreover, feedback from CAB members suggest that they did not want guidelines that would lead to micro-managing applicants and wanted the RFA designed to allow for maximal creativity in scope and design of projects.

The final decision regarding which pilot grant application was funded was the responsibility of the CAB. The Project UNITED’s academic investigators independently evaluated submitted applications to assess the concordance in application scores between academic scholars and non-academic community scholars, but did not have authority regarding the final funding decision. Project UNITED’s academic investigators did assist the CAB in a scholarly capacity, which appeared beneficial when crafting the Institutional Review Board (IRB) oversight statement, non-academic grant reviewer sheet, and additional scientifically rigorous components of the RFA. Additional details about the demands from CAB members during the crafting of the RFA will be detailed in a forthcoming manuscript, but the application review by the CAB and Project UNITED’s investigators produced a consensus ranking regarding which submitted applications should be funded.

### 2.2. Community Engagement to Facilitate the Submittal of CBPR Grant Applications

Social frustrations between academic researchers and non-academic community members leading to wasted financial resources in CBPR projects is a well-documented concern in the literature [20,30]. To combat this challenge, Project UNITED developed and implemented activities designed to promote cultural competency among academic researchers (*n =* 10), research literacy among non-academic community scholars (*n =* 11), and productive scholarly relationships among all participants. The final deliverable of this nine-month program was the submission of a CBPR grant application addressing obesity, which was collectively designed by at least one academic researcher and non-academic community scholar from Alabama’s Black Belt. This section of the manuscript will briefly highlight several CBPR activities designed to promote scholarly engagement between academic researchers and non-academic community scholars. This includes a summary of the: (1) application process to participate in Project UNITED; (2) mini-research school; (3) *CBPR Speed Dating* and scholarly group formation; and (4) Project UNITED’s *e-Chats* and writing retreats.

#### 2.2.1. Application to Participate in Project UNITED

Given the “publish or perish” [31] and hyper-competitive grant funding environment in academia, Project UNITED’s PIs and CAB felt that it was important that program participants were genuinely vested in community health and interested in doing so through community collaborations. Therefore, prospective Project UNITED participants, which include both academic researchers and non-academic community members, were required to formally apply to participate in Project UNITED. Prospective participants submitted their resume/curriculum vitae and responded to essay questions regarding community engagement. These applications were reviewed by the CAB and academic PIs. The opportunity was advertised to academic scholars through email LISTSERVs and university news sources. Non-academic community scholars learned of the opportunity through word of mouth and physical flyers, as well as via personal invitations in keeping with findings from the existing CBPR literature suggesting that it takes the “right kind of community person” to make a scholarly collaboration successful [27]. This application process resulted in 10 academic researchers and 11 non-academic community scholars in Project UNITED’s initial cohort.

#### 2.2.2. Mini-Research School

The first major Project UNITED engagement activity was the mini-research school. The mini-research school was a two-day event hosted over a weekend, which included instructional seminars, discussion forums, and other activities. The mini-research school was designed to promote research literacy among non-academic community participants and cultural competency among academic participants, as well as lay the initial ground work for scholarly collaborations between academic and non-academic participants. Non-academic participants received structured seminars and lectures related to research and important components of its process, while academic participants’ received a comprehensively planned curriculum on cultural competency. To promote cultural competency beyond the realms of a traditional educational setting, Project UNITED believed that it was important for academic participants to spend significant time in a Black Belt community, thereby visually internalizing many of the factors that they had read about in the literature. Project UNITED and its CAB felt that personally witnessing the social and environmental conditions of the Black Belt region informs academic researchers about rural community members in a richer context that what few research articles could provide. Therefore, all activities in the two-day mini-research school were held in a Black Belt community.

For the morning of the first day of the mini-research school, non-academic participants were provided an overview of the fundamental concepts of community-based and health disparities research. In this fundamentals seminar, participants were introduced to current trends in obesity, the definition of health disparities and community-based approaches, as well as current methodologies and approaches to abate health disparities in the CBPR literature. Thereafter, both academic and non-academic participants took a tour of a Black Belt community affected by limited health care access, obesity, and poverty. During this tour, non-academic participants and additional Black Belt community leaders provided first-hand accounts of the deleterious issues affecting their community. This tour had an informal structure, allowing academic scholar participants the opportunity to ask community leaders questions related to issues discussed in the scientific literature. Beyond the information discussed in the tour, this event was important because it formally established community members as the “experts” in a topic area designated as important to academic participants’ education.

After the two-hour tour, all Project UNITED participants, PIs, investigators, and the CAB participated in a lunch and social mixer that served as an ice-breaker for the academic and community engagement process. This social mixer allowed academic and non-academic scholars to get to know each other in a non-professional capacity. Though the relationship between cohesion and performance (*i.e.*, productivity) is complex, cohesion and performance is typically considered to be positively related [32] (*i.e.*, increased levels of cohesion is associated with increased productivity among groups). Hence, the social mixer was an opportunity for participants to potentially gauge similarities and differences (*i.e.*, estimate potential level of cohesiveness) from other participants through discussion about interests and activities related to and outside of health research. After the mixer, participants attended a Black Belt community health fair that informed non-academic participants about various health topics pertinent to population health. The health fair was followed by a one-hour seminar on cultural competency, where (again) all participants were involved, but non-academic participants and other community leaders took the lead to educate academic participants. More specifically, this seminar discussed cultural competency issues germane to community-research. The utility of participatory research was heavily emphasized during this seminar. For the final event of the first day of the mini-research school, academic and non-academic scholars participated in a *CBPR Speed Dating Event*, which will be highlighted in more detail in a later section of this manuscript.

The first seminar of the second day of the mini research school involved a two-hour training session on the academic grant writing process. Though both academic and non-academic participants were involved in this event, this seminar was primarily designed to inform non-academic scholars on the scientific rigor of typical research-oriented grants. For the final event of the mini-research school, non-academic participants attended a two hour seminar on research ethics, human subject’s research, and the purpose and requirements of the institutional research board. All activities were assessed using the first three levels of Kirkpatrick’s hierarchy of behavioral change, namely: (1) Participation, reaction, and satisfaction—how participants feel about aspects of the program or interventions; (2) Learning (knowledge and skills)—knowledge acquired, skills improved, and/or attitudes changed; and (3) Application and implementation (behavior)—a measure of the extent to which participants practice behaviors. The fourth level of this taxonomy describes impact, which is currently being assessed and will be presented in future publications [33].

#### 2.2.3. *CBPR Speed Dating* and Scholarly Group Formation

As a component of the mini-research school, Project UNITED’s academic and non-academic scholars participated in an engagement event labeled *CBPR Speed Dating*. *CBPR Speed Dating* was developed to promote the establishment of scholarly relationships based on common research interests between researchers and community stakeholders prior to the development and execution of CBPR projects. Project UNITED developed *CBPR Speed Dating* with the assumption that the process of fostering productive scholarly relationships between stakeholders is much like a social relationship that has to develop organically. It was Project UNITED’s ultimate goal to promote the neutralization of differences between academic researchers and community leaders early in the CBPR developmental process.

The *CBPR Speed Dating* event took place in a Black Belt high school science lab that provided an ideal environment for frequent movements and simultaneous transitions for 30 individuals with relative ease. During short-timed stationary periods, academic and non-academic scholars were positioned directly across from each other, face-to-face, to facilitate eye contact between participants, so that each party would have a better understanding if the other party was listening. To ensure that the objectives of the *CBPR Speed Dating* activity were clear, topics of discussion were written on a whiteboard (e.g., “What do you envision as a potential strategy to improve obesity prevalence in your community?”).

During *CBPR Speed Dating*, non-academic scholars remained seated, while academic scholars transitioned between non-academic scholars every 10 min. The *CBPR Speed Dating* moderators did their best to limit discussions to 10 min though participants at times went beyond planned time limits. During these 10-min interactions, participants were allowed to exchange contact information (e.g., business cards, resumes) should they desire to remain in contact after the event. In between the 10-min discussions, participants were given approximately two minutes to move to the next table. The *CBPR Speed Dating* event ended once all non-academic participants had the opportunity to spend 10-min with all academic researchers.

After the mini-research school, Project UNITED’s investigators continued to facilitate communications between the academic and non-academic scholars by sharing each participant’s research interests with other participants via email, phone conversations, online forums, and face-to-face meetings. Video biographies were also created so that participants could learn about each other via the Internet at their own convenience. From feedback provided during phone and online conversations, Project UNITED’s investigators identified five topic areas of interest associated with curbing obesity in rural communities, which served as discussion topics for forming community/academic teams among participants. Thereafter, using an online survey, participants were asked to select three Project UNITED participants they wished to work with on a research project. Accordingly, each academic researcher selected three community scholars, and each community scholar selected three academic researchers. Online voting results suggested that most community scholars wanted to work with the sole African American academic researcher, while most academic researchers desired to work with the community scholar that resided in a community that was in close proximity of the academic researchers’ university. Given the challenge that these results provided, Project UNITED’s investigators used biographies and the *CBPR Speed Dating* activity as reminder cues, which were believed to have helped Project UNITED facilitate group formation during a “grant idea” writing retreat with relative ease (this topic is highlighted later in this manuscript).

#### 2.2.4. Project UNITED’s *e-Chats* and Writing Retreats

When providing CBPR training programs for community members, location and availability arose as key factors. In contrast to most academic faculty, volunteering to lead research can be challenging for community leaders with numerous responsibilities that often extend beyond their careers (e.g., social organizations, local “part-time” elected positions, *etc.*). Therefore, while CBPR training for academic faculty is often offered as instructional workshops at academic institutions, special concessions must be considered for training their non-academic counterparts in rural communities given availability and potential travel challenges.

Project UNITED decided that it would be best if non-academic participants could complete their comprehensive CBPR training via a web-based discussion board for their convenience without a compromise in the quality of training. In summary, non-academic scholars participated in eight web-based discussions centered on the CBPR research process. Prior to each session, non-academic participants were sent an email reminding them about the discussion time and the topic that would be discussed. Also, within the email, non-academic participants received short reading assignments intended to enhance the on-line discussions. CBPR topics for discussion were selected by Project UNITED facilitators and the online training sessions focused on: (a) community empowerment; (b) bioethics; (c) translational science; (d) minority health; (e) collaboration strength; (f) collaboration barrier; (g) research interest; and (h) research questions. Though topics were pre-determined, the facilitator welcomed flexibility and allowed web conversations to drift into other research methodological areas of interest. These one-hour online sessions were conducted every two weeks for four months. After the one-hour sessions ended, participants were still able to post comments on discussion boards, which other non-academic participants were able to respond.

In contrast, academic participants received 10 two-hour CBPR-focused lectures in an academic lecture hall. Academic participants’ comprehensive CBPR training covered comparable topics such as: (a) the key principles of CBP; (b) developing a CBPR partnership; (c) developing a CBPR structure; (d) trust and communication; (e) securing and distributing fund; and (f) disseminating CBPR results and CBPR sustainability. Academic participants consistently rated the format, content, and instructor effectiveness for these sessions extremely high. Though emails were exchanged between sessions to gain clarification, academic participants had the opportunity to address their questions in a face-to-face format. Though non-academic participants could have benefited from this educational format, feedback suggests that the convenience of not having to travel appeared to outweigh the benefits of face-to-face instruction.

After the completion of non-academic participants’ training, two writing retreats were scheduled to complete collaborative group formation; and provide appropriate time for groups to write a significant portion of the research grant. The first writing retreat occurred as a one-day event at a regional academic institution. During the first writing retreat, four CBPR collaborative groups were formed from the 20 total participants (one non-academic participant did not complete the program). Most groups were comprised with at least two academic participants and two non-academic participants. During the first writing retreat, ideas for the grant proposals’ specific aims were discussed within groups. Project UNITED mentioned that group applications would be considered for the pilot grant RFA offered by the Project UNITED/CAB (discussed earlier in this manuscript) or another desirable CBPR pilot grant. All groups decided to pursue the Project UNITED/CAB pilot grant with the intention of submitting their application to another funding agency if they did not acquire the internal pilot grant. During this writing retreat, Project UNITED investigators and guest lecturers served as scholarly resources for groups in need of assistance.

The goal of the second one-day writing retreat, several months later, was for collaborative groups to have at least finalized their specific aims, methodology, and the delegation of responsibilities for other components of the CBPR grant application that needed to be completed. This productive one-day retreat was scheduled two-months prior to the Project UNITED/CAB pilot grant application deadline. Therefore, it was not expected that the final application would be completed during this specific event, but having the application’s aims and methodology sections complete allowed sufficient time for collaborative groups to focus on the aspects of grantsmanship that are important for increasing the odds of acquiring funding [34]. The evaluation team for Project UNITED presented information to the groups about the importance of rigorous evaluative measures. Presenters introduced the concept of programmatic evaluation and discussed the use of logic models and SMART objectives (*i.e.*, specific, measurable, assignable, realistic, and time-related objectives). Prior to the end of the retreat, evaluators provided consultation to each group to discuss their intended projects and identify strategies to strengthen their applications.

## 3. Results: Funded Projects Produced by Project UNITED’s Participants

Among the four collaborative groups developed through Project UNITED’s CBPR engagement activities, three (75%) completed and successfully submitted a proposal to fund a pilot study addressing obesity related disparities in a rural community. As of the writing of this manuscript, two collaborative groups have successfully been awarded a grant. One group was awarded the CAB/Project UNITED pilot grant, while the other received funds from another institutional seed funding source. This section will now highlight details of the two-funded proposals.

### 3.1. Home Sweet Home

*Home Sweet Home* is a CBPR project taking place in Greene and Sumter Counties, Alabama. This project was the recipient of Project UNITED and its CAB internal pilot grant award. *Home Sweet Home* is focused on developing a multigenerational childhood obesity prevention program for rural residents. Five of the seven census tracts within these counties are classified as food deserts. The intervention for this project includes curriculum and activities aimed at ameliorating obesity through addressing the collective physical and psychosocial home environment within a rural household, as well as individual level activities designed to boost self-control of risky dietary behaviors, knowledge, and self-efficacy to promote health. This multi-generational health education intervention includes grandparents, parents, guardians, and youth as target populations. Data collection for this project is nearing completion, and this group intends to apply for a larger grant to examine the reproducibility of their current findings. The specific details of this group’s intervention will be presented by the PIs of this project in a forthcoming manuscript.

### 3.2. Assessing Community Readiness for and Attitudes to Health Promotion and Disease Prevention in Pickens County

Despite not being awarded the Project UNITED and its CAB internal pilot grant this group decided to resubmit their idea to another funding organization. This project was funded by a seed funding program hosted by The University of Alabama Division of Community Affairs. Previous research by this group suggests that there is limited knowledge of Alabama Black Belt communities’ collective level of readiness for or attitudes toward specific health promotion interventions focused on obesity. The purpose of this group’s project is to determine attitudes toward health promotion campaigns among individuals from Pickens County, Alabama and to assess their readiness to prevent obesity. Both standard surveys and structured interviews will be collected as forms of data. Data collection is currently underway for this project.

## 4. Discussion: Several Lessons Learned and Going Forward

Preliminary findings suggest that Project UNITED activities are successful at promoting productive scholarly relationships between researchers and community leaders. These findings are based on both programmatic and observational data. Equally important, collaborative groups that were not successfully funded by Project UNITED continue to work together, which led to one additional group acquiring funding from another source. As many academic researchers know, psychological resiliency is paramount in the research grant writing environment.

Though there were successes, like many previous endeavors in the CBPR literature, Project UNITED experienced its challenges. First, one non-academic scholar did not complete the program. Though Project UNITED and the CAB did their best to educate non-academic participants on the process of scientific grant funding, social frustrations were expressed as to why groups had to apply for funds if Project UNITED and the CAB were already provided funds by a government agency. The one participant who did not complete the program conveyed feelings of mistrust related to the handling of research funds. This highlights one disparity often seen between non-profit community project-based funding and research funding. To that extent, Project UNITED will spend more time on educating non-academic scholars on the purpose of biomedical scientific grants in the future.

Second, one of the four groups did not successfully submit a grant application. More specifically, evidence from communications suggests that the group dissolved before the deadline of the Project UNITED pilot grant. As with many social relationships, group dynamics are sometimes not successful, and goals unfortunately cannot be carried out as originally planned. The group that did not successfully submit an application was comprised of bench scientists as academic scholar representatives. The non-academic scholars within this group appeared to have had difficulty in grasping some of the research ideas proposed by their academic counterparts.

Third, many community scholars selected the same African American academic researcher as their first option for research collaborations. The community scholars were all from predominantly African American communities, while the academic scholars were, with one exception, not African American. Though Project UNITED believed that planned activities made this issue easier to handle, it still required constant communication between participants to remind them of similarities in research interests. Project UNITED is exploring the utility of implementing team building activities for participants prior to group formation for future cohorts. This is being explored to determine if activities designed to directly promote cohesion can increase the likelihood that African American community participants will willingly collaborate with White academic participants with minimal interpersonal approaches of group forming from Project UNITED investigators.

Finally, several academic scholars with heavy teaching and administrative responsibilities expressed frustrations with having to travel to rural communities. However, without face-to-face interactions, it is difficult for partners to develop collaborative grant applications and more importantly, strengthen their working relationship with one another. Given this barrier and its implications, interactive video web technologies will be explored to facilitate group meetings in the future.

Future activities for Project UNITED will involve producing more manuscripts further detailing the activities presented in this paper (such as measures, forms and lesson plans as supplementary materials) so that other CBPR scholars can determine the reproducibility of Project UNITED’s successes and lessons learned. Now that relationships are established, Project UNITED also plans to enroll another cohort of participants in the near future after all evaluative activities are complete.

## 5. Conclusions

Our preliminary findings suggest that Project UNITED is on track to facilitate community empowerment through CBPR activities in high obesity risk rural areas. Moreover, we believe that activities researched and implemented in Project UNITED may be an effective model to build the evidence base for CBPR engagement practices, so that community interventions that involve validated forms of treatment will achieve optimal success. Though there is still a lot of work to be done to meet the goals of Project UNITED, we believe that the progress made thus far is promising. In conclusion, fostering collaborations between community leaders and academic faculty is challenging, but if done properly using well-designed CBPR approaches, those collaborations can reap the rewards of community empowerment and sustainable research.
